# Novel Calicivirus Identified in Rabbits, Michigan, USA

**DOI:** 10.3201/eid1512.090839

**Published:** 2009-12

**Authors:** Ingrid L. Bergin, Annabel G. Wise, Steven R. Bolin, Thomas P. Mullaney, Matti Kiupel, Roger K. Maes

**Affiliations:** University of Michigan, Ann Arbor, Michigan, USA (I.L. Bergin); Michigan State University, Lansing, Michigan, USA (A.G. Wise, S.R. Bolin, T.P. Mullaney, M. Kiupel, R.K. Maes)

**Keywords:** Calicivirus, rabbit hemorrhagic disease, Lagovirus, viruses, Michigan, USA, research

## Abstract

We report a disease outbreak in a Michigan rabbitry of a rabbit calicivirus distinct from the foreign animal disease agent, rabbit hemorrhagic disease virus (RHDV). The novel virus has been designated Michigan rabbit calicivirus (MRCV). Caliciviruses of the *Lagovirus* genus other than RHDV have not been described in US rabbit populations. The case-fatality rate was 32.5% (65/200). Clinical signs included hemorrhage and sudden death, with hepatic necrosis. Analysis of viral RNA sequence from >95% of the viral genome showed an average similarity of 79% with RHDV. Similarity of the predicted MRCV capsid amino acid sequence ranged from 89.8% to 91.3%, much lower than the 98% amino acid similarity between RHDV strains. Experimentally infected rabbits lacked clinical disease, but MRCV was detected in tissues by PCR. We propose that MRCV primarily causes subclinical infection but may induce overt RHD-like disease under certain field conditions.

## Abstract 2

This virus is distinct from rabbit hemorrhagic disease virus.

Rabbit hemorrhagic disease (RHD) is caused by a calicivirus and is associated with illness and death in up to 90%–100% of susceptible rabbit populations (*1*–*3*). Domestic rabbits (*Oryctolagus cuniculi*) and free-ranging European rabbits (*O. cunicuusi*) are highly susceptible; cottontail rabbits (*Sylvilagus floridanus*) and hares (*Lepus* spp.) are unaffected. Rabbit hemorrhagic disease virus (RHDV) was first detected in China in 1984 (*4*) and subsequently has been described in eastern and western Europe, Asia, South America, Australia, Mexico, and the United Kingdom (*2*,*3*). In the United States, it is considered a foreign animal disease, and outbreaks are of considerable economic concern to the US rabbit industry. In the United States 4 outbreaks of RHD have occurred in domestic, captive rabbits (*O. cuniculi*) since 2000. These cases were confirmed by inoculation study and reverse transcription–PCR (RT-PCR) because the virus is not cultivable in vitro. Subsequent genomic analyses suggested that these incidences resulted from separate viral introductions; however, confirmed points of origin (e.g., imported animal or animal product) were never identified (*3*).

RHDV is classified as a *Lagovirus* within the family *Caliciviridae*. *Caliciviridae* are nonenveloped, positive-sense, single-strand RNA viruses. Within this family are 4 genera: *Lagovirus, Vesivirus, Norovirus*, and *Sapovirus* (*5*). In addition to the highly pathogenic RHDV, the *Lagovirus* genus includes several distinct but related viruses affecting rabbits or hares. These are European Brown hare syndrome virus (EBHSV), which causes disease similar to RHDV in hares only (*Lepus* spp.) (*6*), and the nonpathogenic rabbit calicivirus (RCV), which causes asymptomatic seroconversion in rabbits (*7*). RCV and EBHSV have not been reported and lagoviruses other than RHDV have not been described in US rabbit populations, although a low pathogenicity rabbit *Vesivirus* recently was identified in domestic rabbits from Oregon (*8*). RHDV affects only rabbits of the *Oryctolagus* genus which, in the United States, is limited to domestic rabbit species. Wild rabbit species in the United States are not experimentally susceptible (*9*). Seroprevalence surveys of RHDV or related caliciviruses in US domestic rabbit populations have not, to our knowledge, been published.

Beginning January 1, 2001, and continuing over a 3-week period, a privately owned New Zealand White (*O. cuniculi*) rabbitry in Michigan experienced acute fatalities. Before this episode, the farm had operated for 1.5 years without notable disease. Approximately 200 rabbits were kept in a closed barn on a 60-acre farm; new rabbits had not been acquired in 18 months. Inappetence in several animals and vulvar hemorrhage in pregnant does were initially noted. A total of 65 rabbits—consisting of 23 adult does (most pregnant), 2 adult bucks, and 41 young rabbits of both sexes—died over ≈3 weeks, for a case-fatality rate of 32.5%. Clinical signs consisted of vulvar hemorrhage in the does, epistaxis, ataxia, opisthotonos, diarrhea, ocular discharge, vocalization, and death.

## Materials and Methods

### Biological Samples

Three pregnant does (2 live) with vulvar hemorrhage were submitted for diagnostic evaluation on January 3. Eighteen additional rabbits of either sex ranging from 2 through 9 months of age were submitted near the end of the disease outbreak on January 17 and February 2. Animals submitted live were humanely euthanized by intravenous or intraperitoneal injection of pentobarbital (Fatal-Plus, Vortech, Dearborn, MI, USA) at 1 mL per 10 pounds.

Tissue samples from representative organs of all submitted rabbits were immersion fixed in 10% neutral buffered formalin. We processed tissue samples for histopathology using standard methods. Transmission electron microscopy was performed on a negatively stained clarified pooled liver homogenate from 2 of the originally submitted does using standard procedures.

For immunohistochemistry, liver sections from an affected rabbit were deparaffinized and rehydrated by routine methods. Antigen retrieval was by proteinase K (Dako, Carpinteria, CA, USA) for 10 min at 37°C. Endogenous peroxidase was blocked for 15 min with 3% hydrogen peroxide, and nonspecific immunoglobulin binding was blocked by 10 min incubation with a protein-blocking agent (Dako). Guinea pig anti-RHDV VP60 antibody (dilution 1:1,000) provided by Dr. F. Parra (*10*) was applied for 30 min at room temperature in a Dako autostainer. After incubation with rabbit anti–guinea pig immunoglobulin (Ig) G, detection was by a chain polymer conjugated staining procedure (EnVision, Dako) with visualization by AEC (Dako) and Mayer’s hematoxylin counterstaining. Negative controls were homologous (guinea pig) nonimmune serum applied to rabbit liver sections.

### In Situ Hybridization

In situ hybridization was performed as described (*11*). Briefly, liver sections from an affected and an experimental rabbit were deparaffinized, digested with 0.25% pepsin and prehybridized. Hybridization was for 5 min at 105°C and 60 min at 37°C with a specific 3′-end digoxigenin-labeled oligoprobe (5′-GAGAGTCGTCTCGGTAGTACCTG-3′, IDT, Coralville, IA, USA) at 5 μL/1 mL using a commercial workstation (Fischer Scientific, Pittsburgh, PA, USA). Detection was by antidigoxigenin (Boehringer Mannheim Biochemica, Indianapolis, IN, USA) conjugated with alkaline phosphatase (dilution 1:500, Boehringer Mannheim Biochemica) and the substrates NBT/X-Phos (nitro-blue tetrazolium/5-bromo-4-chloro-3-indolylphosphate, Boehringer Mannheim Biochemica). A non-sense probe was applied to liver sections as negative control.

### Bacterial Cultures

Representative tissues from the original 3 does and 14 subsequently submitted rabbits were cultured by using standard microbiologic techniques. Virus isolation was attempted by injection of filtered (0.45-µm pore size) 10% homogenate of liver (pooled from 2 of the originally submitted does) in Bovarnik’s buffer onto cultures of RK-13, R9ab, or SIRC cells.

### Nucleic Acid Extraction and PCR

Total RNA from pooled liver of 2 initially submitted does was extracted with RNeasy Mini Kit (QIAGEN, Inc., Valencia, CA, USA), according to the manufacturer’s guidelines. A 398-bp region of the VP60 (capsid) gene was initially targeted using RHDV-specific primers (*12*). RT-PCR was performed on total liver RNA using a One-Step RT-PCR Kit (QIAGEN) with forward and reverse primers, 5′-GTT ACG ACT GTG CAG GCC TAT GAG TT-3′ and 5′-TTG TTG AGC AGT CCA ATT GTC ACT G-3′, respectively, at 0.6 μM in a 50-μL reaction. Cycling conditions were as follows: cDNA synthesis at 50°C for 30 min; then predenaturation at 95°C for 15 min and 43 cycles of 94°C for 30 sec, 55°C for 30 sec, and 72°C for 1 min; and final extension of 72°C for 10 min. Products were visualized in prestained agarose gels by a UV transillumination. The entire capsid gene (≈1.8 kb) was amplified by using primers based on USA Iowa 2000 RHDV strain (spanning nucleotide positions 5273–7065, GenBank accession no. AF258618). Forward and reverse primers, 5′-CGG TAG TAC CTG ACG ACG AAT TTG-3′ and 5′-GCA AGT CCC AGT CCG ATG AAT-3′, respectively, were used as above, with the modification of 35 cycles with 2 min of extension. Further genomic sequencing used primers from conserved RHDV and Michigan rabbit calicivirus (MRCV)–derived sequences (Table) with annealing temperatures 52°C–55°C and extension times 1 min per 1-kb target length. MRCV genomic ends were sequenced using 5′ and 3′ RACE Systems for Rapid Amplification of cDNA Ends (Invitrogen, Carlsbad, CA, USA). For 5′ RACE, cDNA was synthesized with gene-specific primer, GSP1, 5′-ACT GTA CTC CCT GGG TGC GAC-3′ (MRCV, 2039–2059, minus sense) with purification and dCTP-tailing with TdT. The dC-tailed cDNA was PCR amplified by using HotStar Taq DNA Polymerase Kit (QIAGEN) in a 50-µL reaction with 5 µL dC-tailed cDNA, 0.5 µM abridged anchor primer, and gene-specific primer (GSP2) 5′-CAT CGC CGC TGG TGT TAA ACT-3′ (MRCV, 1662–1682, minus sense) at 95°C for 15 min and 35–45 cycles of 94°C for 30 sec, 55°C for 30 sec, and 72°C for 2 min 30 sec; with final extension 72°C for 7 min. For 3′ RACE, cDNA was synthesized with an oligo-dT-containing adapter primer according to the kit manufacturer’s protocol. PCR was performed with gene-specific primer, 5′-GTT ACG ACT GTG CAG GCC TAT GAG TT-3′ (the same used in the initial RHDV-specific PCR) (*12*) and the kit component abridged universal primer with reaction specifications above.

**Table:** Primers used in genomic sequencing of Michigan rabbit calicivirus*

Genomic region(s) spanned	Forward and reverse primer pairs†	Nucleotide positions in RHDV‡
Polymerase-capsid (VP60)	5′-ATGCCATGACTCCGATGATGGT-3′ (+) R	4835–4856
	5′-CTTGTTGGTCCACCTGTTG-3′ (−) M	5482–5500
Polymerase	5′- GCGACTTCTTGTGCTTGGACTAC-3′ (+) R	4493–4515
	5′- GCACCACTCCAACTGTCTGAGAA-3′ (−) M	5027–5049
Polymerase	5′-GTGACCCAGACAGTGACAAGT-3′ (+) R	3929–3949
	5′-GGCCTATTTCTGCACATGCTT-3′ (−) M	4754–4774
Protease-polymerase	5′-GCGGTGACCARGGTGTTGATG-3′ (+) R	2819–2839
	5′-GCCGCAGCACGCTCTATGAAT-3′ (−) M	4020–4040
VPg-protease	5′-AACAAAGCCGTTGAAAGTTGG-3′ (+) R	1927–1947
	5′-TGGCAGCTCTGTTCTTCATTT-3′ (−) M	3119–3139
Helicase	5′-GAGGTTGTTTGACACGTTTGA-3′ (+) R	1089–1109
	5′-TGTCATATTCACACAGCCCAG -3′ (−) M	2398–2418
Capsid (VP60)–ORF 2	5′-GTTACGACAGTGCAGGCCTATG-3′ (+) M	6412–6433
	5′-CTCGCCAGTGGTGTTATAAATC -3′ (−) R	7345–7366

*RHDV, rabbit hemorrhagic disease virus; MRCV, Michigan rabbit calicivirus; VP, viral protein; ORF, open reading frame. †RHDV-specific primer denoted by R; MRCV-specific primer denoted by M. ‡Positions spanned by primer set in RHDV genome (Italy BS89 strain, GenBank accession no. X87607).

### Sequencing and Sequence Analyses

RT-PCR amplicons were purified from 1.4% agarose gels by QIAquick Gel Extraction Kit (QIAGEN). PCR products were sequenced by the Research Technology Support Facility of Michigan State University by automated DNA sequencing on an ABI Prism 3100 Genetic Analyzer (Applied Biosystems, Foster City, CA, USA). Long PCR templates were sequenced by using primer-walking with newly synthesized 5′ and 3′ primers derived with OLIGO 6 primer analysis software (Molecular Biology Insights, Cascade, CO, USA). Similar GenBank sequences were detected by BLAST analysis (*13*). Sequence assembly and analyses, including multiple Clustal W (www.ebi.ac.uk/clustalw) sequence alignments and construction of phylogeny trees, were done with Lasergene software (DNASTAR, Inc., Madison, WI, USA). GenBank accession numbers for complete and partial rabbit caliciviral nucleotide sequences used for phylogenetic analysis are as follows: RHDV strains USA IA00 (AF258618), USA UT01 (EU003582), USA NY01 (EU003581), USA IN05 (EU003579), JX China 97 (DQ205345), China CD (AY523410), China WHNRH (DQ280493), Italy 90 (EU003580), Germany FRG (NC001543), China WX84 (AF402614), Mexico 89 (AF295785), Spain AST89 (Z49271), Ireland 18 (DQ367359), Italy BS89(X87607), EBHSV (NC002615), RCV (X96868), MRCV (GQ166866).

### Inoculation Study

Fourteen 16-week-old male and female, specific pathogen–free New Zealand White rabbits (Harlan Sprague Dawley, Inc., Indianapolis, IN, USA) were each inoculated intramuscularly, orally, and intranasally with 2 mL total of liver homogenate containing 10^4^–10^5^ PCR detectable units of viral RNA per mL, as determined by PCR assay on serial 10-fold dilutions of RNA extracted from the inoculum. Homogenate was prepared from frozen (−80ºC) liver from 2 of the originally submitted does. Two control rabbits were in containment housing in the same cage bank but not in direct contact with inoculated rabbits and were not sham inoculated. The rabbits were free of *Pasteurella multocida, Bordetella bronchiseptica, Encephalitozoon cuniculi, Treponema cuniculi, Clostridium piliforme,* myxomatosis virus, RHDV, *Toxoplasma* spp., and coccidiosis. Animal husbandry was in keeping with the Animal Welfare Act and US Public Health Service policy, and the infection study protocol was approved by the University Animal Care and Use Committee.

Rabbits were monitored for clinical changes and were humanely euthanatized by intravenous pentobarbital injection after 2 (n = 2 control rabbits), 4 (n = 2), or 7 (n = 12) days of infection. Complete necropsies were performed and tissue samples harvested for histology, serology, and RT-PCR. The MRCV capsid encoding region was cloned into a baculovirus expression vector by standard methods, enabling serologic testing by application of serum to baculovirus-expressing insect cells and immunoperoxidase detection.

## Results

### Gross and Histologic Findings

**Figure 1:**
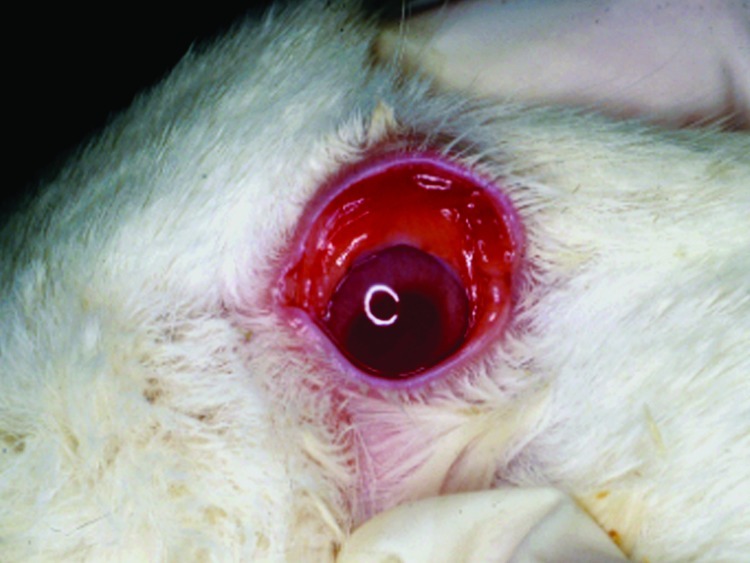
Conjunctival erythema in affected doe.

**Figure 2:**
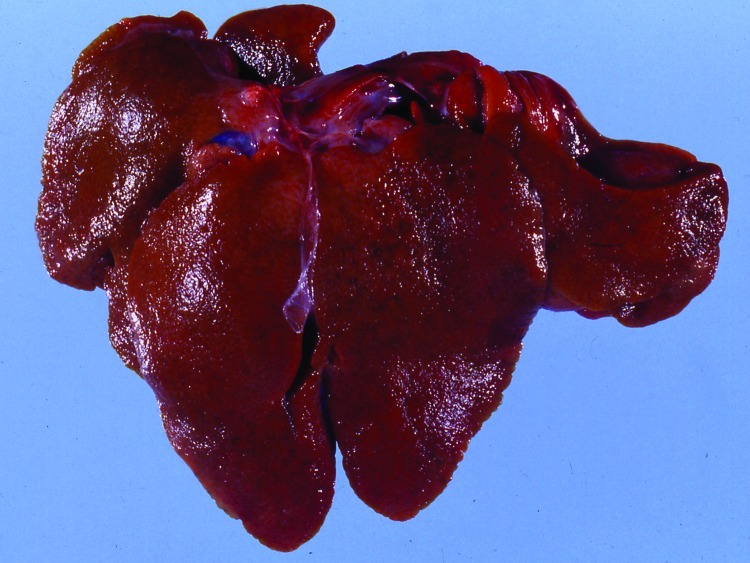
Liver of affected rabbit with granular texture, accentuated lobular pattern, and multifocal capsular petechiae.

**Figure 3:**
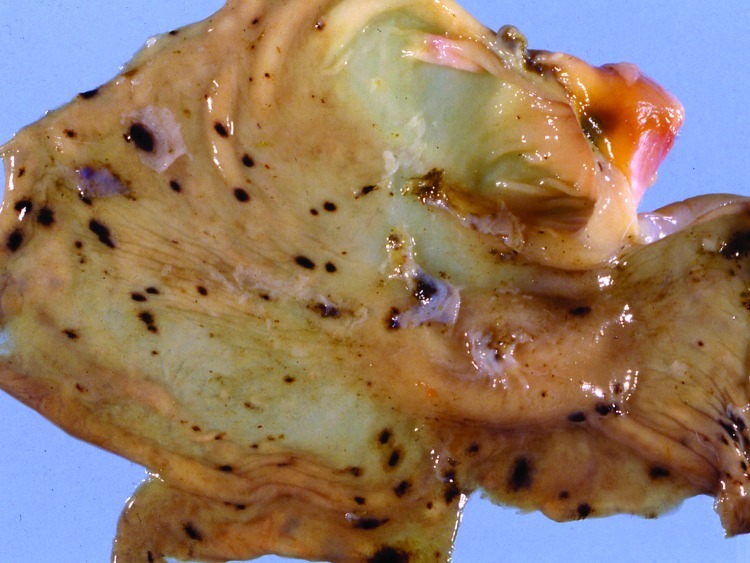
Multifocal gastric hemorrhage in affected rabbit.

The animals submitted for diagnostic evaluation were in good body condition. Several had conjunctival congestion (Figure 1) and mild cyanosis of the lips and ear tips before euthanasia. The pregnant does had small amounts of vulvar hemorrhage and cutaneous hyperemia. The gravid uteri in the does had red to purple serosal discoloration with serosanguinous luminal fluid. Fetuses were in good condition and appeared normal. The livers of all does and 2 of the young adult rabbits were friable and tan and had accentuated lobular pattern (Figure 2). Individual rabbits variably exhibited icterus, opisthotonos, gastric petechiae and ecchymoses (Figure 3), colonic serosal hemorrhage, and multifocal hemorrhage in caudal lung lobes.

**Figure 4:**
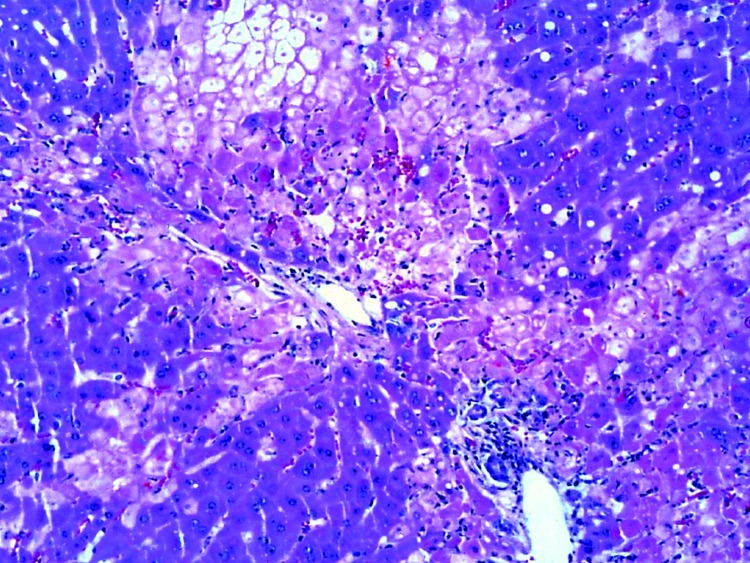
Multifocal periportal and midzonal heptic necrosis in affected rabbit. Hematoxylin and eosin stain. Original magnification ×200.

In the 3 initially submitted does, the major histologic finding was multifocal random or periportal hepatocellular necrosis (Figure 4). Additionally, we found mild periportal heterophilic (neutrophilic) and lymphoplasmacytic inflammation. There were also pulmonary and uterine hemorrhages with fibrin clots in areas of placental implantation. In the 18 subsequently submitted young adult rabbits, predominant histologic findings were moderate expansion of portal tracts with bile duct proliferation, periductal fibrosis, and mild periportal lymphoplasmacytic inflammation. Five rabbits had concurrent heterophilic and bronchopneumonia, and 1 had suppurative meningitis.

### Initial Diagnostic Testing

Caliciviral-like particles were detected in pooled liver homogenate from 2 of the originally affected does by transmission electron microscopy. On request, the US Department of Agriculture Foreign Animal Disease Diagnostic Laboratory further tested tissue samples. Results from inoculation testing and RT-PCR using standard primers (*3*) within the RHDV polymerase region at FADDL were not consistent with RHD, and RHDV was ruled out as the cause of the outbreak.

Uterine cultures from 1 of the initially submitted does grew >1,000 colony-forming units (CFU)/mL *P. multocida*. One young adult rabbit with hepatic necrosis and concurrent bronchopneumonia grew >1,000 CFU/mL *P. multocida* from the lung. Two other rabbits had smaller CFU/mL (<100) *P. multocida* and *B. bronchiseptica* from the lung. Three animals grew low CFU/mL *Escherichia coli* (<100) from the liver. Virus isolation was not successful. Anticoagulants were not detected within liver samples, and no organic toxins were detected by gas chromatography/mass spectrometry.

### Immunohistochemistry and In Situ Hybridization

**Figure 5:**
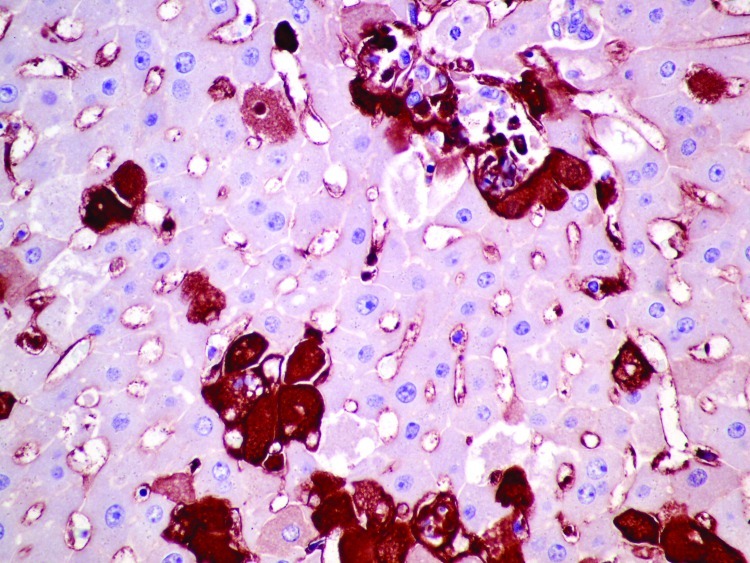
Liver of affected rabbit with positive cytoplasmic immunohistochemical labeling in hepatocytes against rabbit hemorrhagic disease virus capsid. Original magnification ×400.

**Figure 6:**
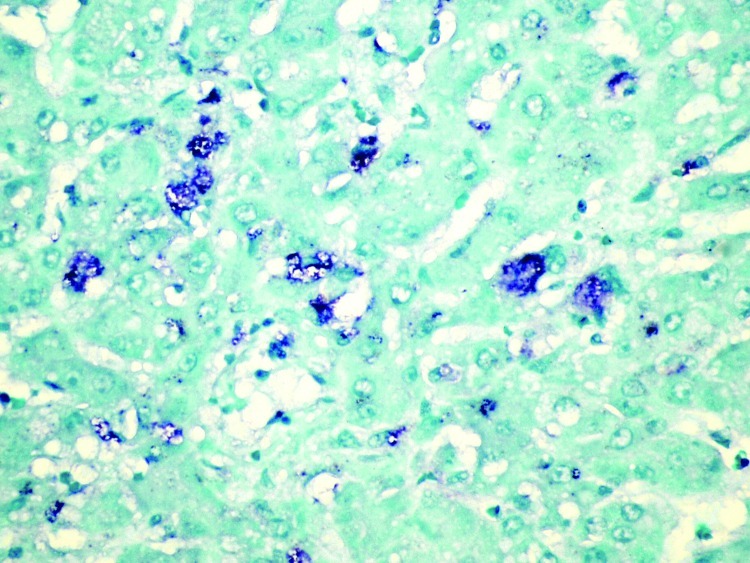
Liver of affected rabbit showing in situ hybridization of a Michigan rabbit calicivirus-specific oligonucleotide probe within scattered hepatocytes. Original magnification ×400.

RHDV was immunohistochemically detected within the cytoplasm of approximately 20% of hepatocytes in 1 of the initially submitted does, primarily in the periportal and midzonal areas (Figure 5). MRCV nucleic acid was detected by in situ hybridization in scattered hepatocytes and few Kupffer cells (Figure 6).

### Capsid Amplification

Using primers targeting the RHDV capsid gene (VP60), we detected a 398-bp amplicon by RT-PCR on pooled liver samples from 2 originally submitted does. BLAST analysis (*13*) of a unique 344-bp sequence (excluding primer sequences) showed average 79% similarity to GenBank RHDV capsid sequences. This unique sequence translates into 113 amino acids with 86.8% similarity to the partial capsid protein in a representative strain of RHDV (FRG, GenBank NC_001543). For comparison, RHDV capsid sequences typically have 98% identity with one another (*7*). Subsequent sequencing was performed on the same pooled liver sample; however, the 398-capsid amplicon was detected in liver and spleen samples from 4 additional clinically affected rabbits.

### Capsid and Genomic Sequencing and Analysis

**Figure 7:**
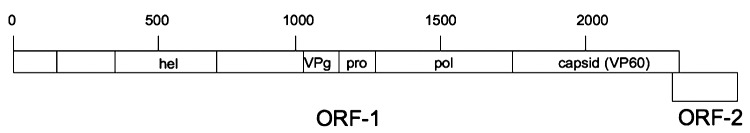
Schematic genomic organization of Michigan rabbit calicivirus consistent with a *Lagovirus* in the family *Caliciviridae*. Lagoviruses contain an initial large open reading frame (ORF), ORF-1 encoding a polypeptide that overlaps with a smaller ORF, ORF-2. Numbering indicates the corresponding amino acid codons predicated from the genomic sequence. hel, helicase; Vpg, virion protein, linked to genome; pro, protease; pol, polymerase; capsid (VP60), capsid protein VP60.

Further amplification of the entire VP60 gene and upstream and downstream regions generated a genomic sequence of 7,387 nt, which is similar to the approximately 7.4-kb length of RHDV (*3*). By alignment with other *Lagovirus* sequences, this included all but 32 bases of the 5′ end of the first open reading frame (ORF) of the genome, which could not be sequenced despite multiple attempts. ORF analysis showed a large (2,328 amino acid) ORF-1 and a smaller (113 amino acid) ORF-2 that overlapped ORF-1 by 8 nt (Figure 7). ORF-1 included conserved sequence motifs for nonstructural (helicases, proteases, and polymerases) and structural proteins (VPg and capsid) in the expected order and location for the *Lagovirus* genus within the *Caliciviridae* family (*5*). ORF-2 included conserved sequence motifs for the secondary structural protein VP12. A novel calicivirus related to but distinct from RHDV was tentatively identified. The isolate was termed MRCV, and the genome was further characterized.

### Multiple Sequence Alignment and Phylogenetic Analysis

**Figure 8:**
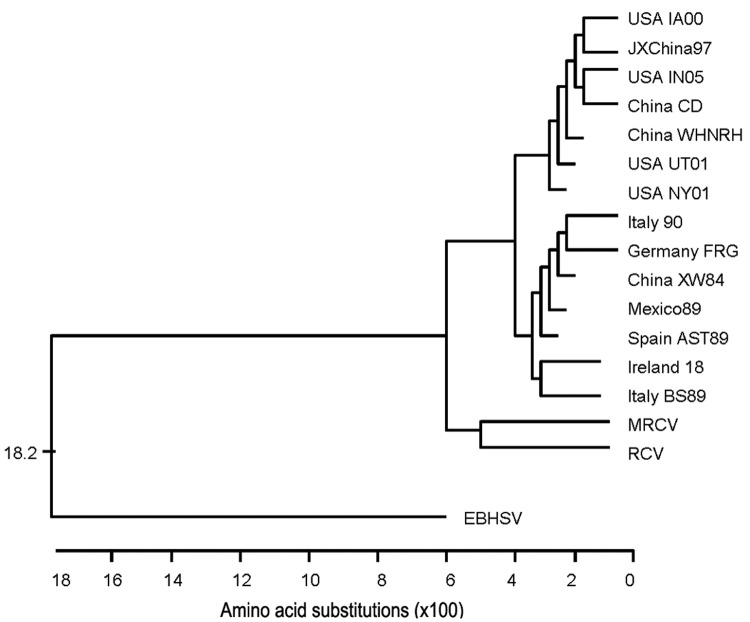
Dendrogram constructed for comparison of capsid (VP60) amino acid sequences. The geographically and numerically named species are strains of rabbit hemorrhagic disease virus. MRCV, Michigan rabbit calicivirus; RCV, rabbit calicivirus.

**Figure 9:**
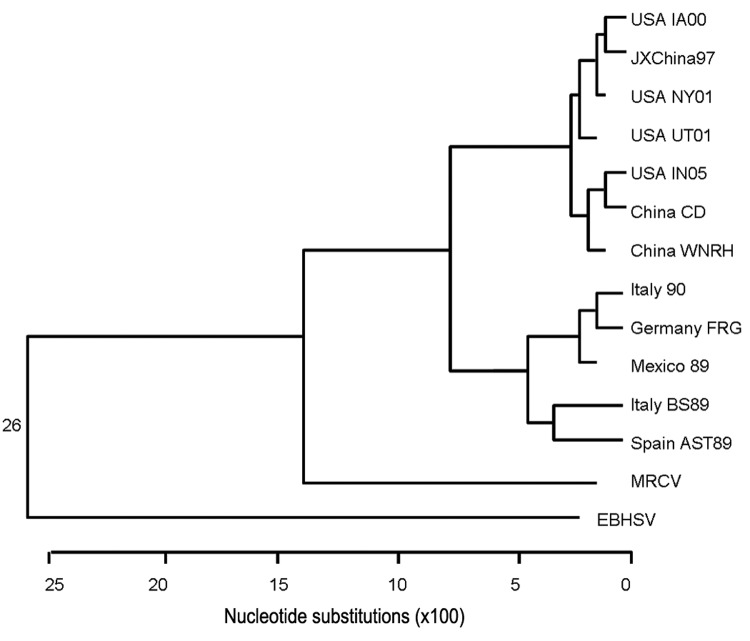
Dendrogram constructed for comparison of open reading frame 1 polypeptide genomic sequence minus the capsid sequence. The geographically and numerically named species are strains of rabbit hemorrhagic disease virus. MRCV, Michigan rabbit calicivirus; EBHSV, European brown hare syndrome virus.

Because most phylogenetic comparisons of RHDV strains are based on capsid sequence, a dendrogram was generated on the basis of MRCV capsid amino acid sequence in comparison with multiple RHDV strains and with the outliers EBSHV and RCV (Figure 8). By this analysis, MRCV appears as an outlier to RHDV strains and is most closely related to the nonpathogenic RCV. MRCV capsid sequence alignments showed 91.7% similarity to RCV and 89.8%–91.3% similarity to RHDV strains. In comparison, RHDV strains shared 95.0%-99.8% similarity with each other and 91.3%–92.7% with RCV. Because the capsid gene may be subject to recombination and positive selection, relatedness predicted solely on capsid sequence is prone to error (*3*). Further analysis compared nucleotide similarities of the ORF-1 polypeptide genomic sequence, excluding the capsid gene (Figure 9). RCV is excluded from this comparison because only capsid sequence is available (*7*). By this analysis, MRCV again appears as an outlier to RHDV, having 77.9%–78.5% similarity to RHDV sequences, which share similarities of 87.8%–98.1% among themselves.

**Figure 10:**
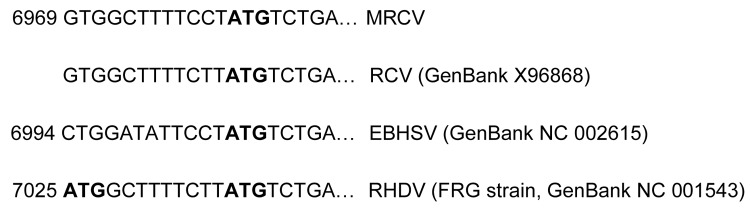
Alignment of open reading frame 2 sequences showing that Michigan rabbit calicivirus (MRCV) follows the pattern of European brown hare syndrome virus (EBHSV) and rabbit calicirus (RCV) in having 1 initiation codon (ATG, in **boldface**) in comparison with the 2 present in rabbit hemorrhagic disease virus (RHDV).

We undertook some additional comparisons of particular genomic regions. The amino acid sequence of MRCV polymerase was only 88.1% homologous to 4 representative RHDV strains, whereas polymerase is typically close to 100% conserved within viral species. Additionally, at the beginning of ORF-2, RHDV strains have 2 initiation codons, and RCV and EBHSV have only 1. MRCV appears to follow the pattern of RCV and EBHSV (Figure 10). These analyses support that MRCV represents a separate, novel caliciviral species.

### Inoculation Study

We attempted to reproduce clinical signs of disease with an inoculation study. Clinical signs in inoculated rabbits were limited to decreased activity and inappetence in 2 rabbits on day 4 postinoculation. These rabbits were euthanized, and necropsy indicated they lacked gross or histologic lesions. The other inoculated rabbits remained free of clinical disease through the end of the study and lacked gross postmortem lesions. Histologically there was rare, individual hepatocellular necrosis. Several tissues, including liver, spleen, stomach, jejunum, ileum, cecum, colon, and (in 3 rabbits) trachea/lung were positive for viral RNA by RT-PCR and in situ hybridization was positive in rare hepatocytes within the liver (data not shown).

### Depopulation and Necropsy Findings in Remaining Rabbits

Further disease in the affected rabbitry was not reported after February 2, and disease outbreaks in additional rabbitries served by the same rabbit dealer were not identified. For economic reasons, the owner of the premises decided to depopulate the remaining rabbits ≈2 months later. Approximately 130 rabbits were submitted for euthanasia. Twenty-five animals had gross changes in their livers, consisting of a granular capsular texture, firm and collapsed tips of ventral lobes, and firm parenchymal nodules up to 2 cm in diameter (data not shown). Representative tissues from 4 animals were evaluated histologically. The liver showed moderate biliary hyperplasia and periductal to bridging portal-to-portal fibrosis. Although these chronic alterations suggest previous liver insult, they are nonspecific with respect to cause.

### Serology

Initial diagnostic evaluations did not include serology because of a lack of reagents, but expression of the viral capsid within a baculovirus system enabled serologic testing by the time of depopulation. Antibody against MRCV was detected in serum harvested from depopulated rabbits (data not shown) but not in any of the experimentally inoculated rabbits that were euthanized as late as 7 days postinoculation.

## Discussion

In this study, a naturally occurring disease outbreak with clinical signs and pathologic findings suggestive of RHD was ultimately associated with a novel rabbit calicivirus, designated MRCV. Although other pathogens, principally *P. multocida*, were identified in some animals, these were not consistently present, and the disease lesions and presentation were not typical of pasteurellosis. Other hemorrhage-inducing agents, such as anticoagulants or enterohemorrhagic *E. coli* (*14*), were not identified. In the animals submitted toward the end of the outbreak, the histologic findings were consistent with a reparative response to a previous liver insult. Similar reparative histologic findings have been identified in rabbits surviving experimental challenge with less virulent strains of RHDV (*15*).

Based on the percentage of affected animals in the outbreak and the failure to generate clinical disease in inoculated specific pathogen–free (SPF) rabbits, MRCV appears likely to be of low pathogenicity, and clinically evident disease may depend on the health status, age, or individual susceptibility of the host. RHDV is known to be most pathogenic in rabbits >8 weeks of age, possibly because of age-related differences in the expression of enterocyte receptors targeted by the virus (*16*–*19*). The symptomatic animals in this report were all >8 weeks of age. Many were pregnant does, and some had concurrent *P. multocida* infection. Although the inoculated rabbits were also >8 weeks of age, they were nonpregnant, commercially available SPF animals and lacked the apparently endemic *P. multocida* present in the diagnostic cases. RHDV-associated illness and death in laboratory rabbits can be lower than in field situations unless the animals are immune-primed by other disease agents (*3*,*15*). Alternately, the failure to reproduce disease may be due to low viable virus dose (difficult to assess for a noncultivable virus) or premature euthanasia of the inoculated rabbits. Although euthanasia by day 7 was consistent with the anticipated timeframe for caliciviral-induced disease, it may not have been sufficient if a low viral dose was used and was likely too early to expect seroconversion.

The phylogenetic analyses presented here indicated that MRCV is a distinct species from RHDV and the other known lagoviruses, RCV and EBHSV. MRCV is the first lagovirus other than RHDV detected in US rabbits; however, seropositivity to low or nonpathogenic caliciviruses has been demonstrated in rabbit populations in several other countries (*7*,*19*–*21*). Additional low pathogenicity strains may exist undetected in US rabbit populations. Samples to consider for RT-PCR detection of rabbit lagoviruses include liver, lung, spleen, and intestine. Although MRCV in this case was detected in liver, other low pathogenicity caliciviruses such as RCV replicate in intestine and may not be detected if liver is the sole sample evaluated (*7*). Infection with low or nonpathogenic lagoviruse*s* in other countries has been postulated to mediate some protection against RHDV infection, although whether this is true of MRCV infection is unknown (*20*,*21*). As evidenced by this case study, diagnosticians faced with hemorrhagic disease and sudden death in rabbits should consider the possibility of low pathogenicity lagoviruses once RHDV has been ruled out. The prevalence and effects of low pathogenicity lagoviruses like MRCV within the US rabbit industry and their potential influence on seropositivity and susceptibility to RHDV remain to be determined.
